# γH2AX Foci Form Preferentially in Euchromatin after Ionising-Radiation

**DOI:** 10.1371/journal.pone.0001057

**Published:** 2007-10-24

**Authors:** Ian G. Cowell, Nicola J. Sunter, Prim B. Singh, Caroline A. Austin, Barbara W. Durkacz, Michael J. Tilby

**Affiliations:** 1 Institute for Cell and Molecular Biosciences, Newcastle University, Newcastle upon Tyne, United Kingdom; 2 Division of Tumour Biology, Forschungszentrum Borstel, Borstel, Germany; 3 Northern Institute for Cancer Research, Newcastle University, Newcastle upon Tyne, United Kingdom; Duke University, United States of America

## Abstract

**Background:**

The histone variant histone H2A.X comprises up to 25% of the H2A complement in mammalian cells. It is rapidly phosphorylated following exposure of cells to double-strand break (DSB) inducing agents such as ionising radiation. Within minutes of DSB generation, H2AX molecules are phosphorylated in large chromatin domains flanking DNA double-strand breaks (DSBs); these domains can be observed by immunofluorescence microscopy and are termed γH2AX foci. H2AX phosphorylation is believed to have a role mounting an efficient cellular response to DNA damage. Theoretical considerations suggest an essentially random chromosomal distribution of X-ray induced DSBs, and experimental evidence does not consistently indicate otherwise. However, we observed an apparently uneven distribution of γH2AX foci following X-irradiation with regions of the nucleus devoid of foci.

**Methodology/Principle Findings:**

Using immunofluorescence microscopy, we show that focal phosphorylation of histone H2AX occurs preferentially in euchromatic regions of the genome following X-irradiation. H2AX phosphorylation has also been demonstrated previously to occur at stalled replication forks induced by UV radiation or exposure to agents such as hydroxyurea. In this study, treatment of S-phase cells with hydroxyurea lead to efficient H2AX phosphorylation in both euchromatin and heterochromatin at times when these chromatin compartments were undergoing replication. This suggests a block to H2AX phosphorylation in heterochromatin that is at least partially relieved by ongoing DNA replication.

**Conclusions/Significance:**

We discus a number of possible mechanisms that could account for the observed pattern of H2AX phosphorylation. Since γH2AX is regarded as forming a platform for the recruitment or retention of other DNA repair and signaling molecules, these findings imply that the processing of DSBs in heterochromatin differs from that in euchromatic regions. The differential responses of heterochromatic and euchromatic compartments of the genome to DSBs will have implications for understanding the processes of DNA repair in relation to nuclear and chromatin organization.

## Introduction

Up to 25% of the histone H2A complement in mammalian cells consists of the histone variant H2AX [1], [2]. Compared to histone H2A1, this molecule has a unique C-terminal tail containing the phosphorylation target sequence for members of the phosphatidylinositol 3′-kinase like kinase (PIKK) family of serine/threonine protein kinases. This family includes ataxia telangiectasia mutated (ATM), ataxia telangiectasia and Rad3 related (ATR) and DNA-dependent protein kinase (DNA-PK)[3], [4]. Histone H2AX is rapidly phosphorylated at Ser139 following treatments that induce DNA double-strand breaks (DSBs) or cause replication stress. At DSBs generated by ionizing radiation for example, H2AX becomes phosphorylated over megabase chromatin regions flanking the breaks [1]. This phosphorylation is dependent largely on ATM, with some redundancy with DNA-PK [5], [6]. The resulting local concentrations of phosphorylated H2AX (γH2AX) can be detected at interphase by immunofluorescence microscopy, and are termed γH2AX foci. UV exposure or treatment with replication inhibitors such as hydroxyurea lead to ATR-dependent H2AX phosphorylation at sites of arrested replication forks [7]. Similarly, replication-dependent DSBs induced by topoisomerase I inhibitors lead to ATR-dependent H2AX phosphorylation [8]. γH2AX is believed to form a platform for the recruitment and/or retention of DNA repair and signaling molecules at sites of DNA damage. At least one of these components, MDC1, binds directly to the phosphorylated C-terminal tail of histone H2AX. The precise physiological role of H2AX phosphorylation is not yet fully understood, but cells derived from H2AX^−/−^ mice display moderate radiosensitivity [9], [10] and a G2/M checkpoint defect [11]. This is consistent with the notion that by concentrating signaling molecules at sites of damage, γH2AX amplifies the DNA damage signal. It has also been suggested that phosphorylation of H2AX helps anchor chromosomal ends together, reducing the chances of DSBs leading to illegitimate recombination events [12].

Phosphorylation of histone H2AX can be seen as one of a number of histone posttranslational modifications that delineate specific functions in particular segments of chromatin. Other such modifications include trimethylation of histone H3 lysine 9 and histone H4 lysine 20, that are characteristic of constitutive heterochromatin [13], [14], [15]. This compartment of the genome is gene-poor and remains condensed during interphase. It is composed largely of repeated elements found in centromeric and pericentromeric regions in most eukaryotes and in the short arms of the human acrocentric chromosomes. DNA replication occurs towards the end of S-phase in heterochromatic regions, whereas euchromatic regions generally replicate in early to mid S-phase. In addition, it is well established that heterochromatic regions are associated with the non-histone chromatin protein, HP1 [16], [17], [18], [19], [20]. Since heterochromatin and euchromatin represent different chromatin environments, it is possible that differences exist in their susceptibility to DNA damage, or in the detection or processing of DSBs. A number of previous papers have examined the frequency of chromosomal abnormalities (CAs) involving euchromatic versus heterochromatic regions following ionizing radiation, as a proxy for DNA damage and repair. No consistent pattern emerges from the literature, possibly because of differences in the species or cell type used or the means by which CAs were examined. Notably though, when Puerto et al (2001) [21] compared the human constitutive heterochromatic 1cen-1q12 region with the similarly sized euchromatic 17cen-p53 region they found no difference in the initial number of γ-radiation induced chromosome breaks, leading to the conclusion that chromatin configuration does not affect radiosensitivity. Histone H2AX phosphorylation is a well established marker of DSBs, and in this study we have found that following ionising radiation, γH2AX foci, are under-represented in heterochromatin in mammalian cells.

## Results

### Ionizing radiation-induced γH2AX foci are largely excluded from heterochromatin

**Figure 1:**
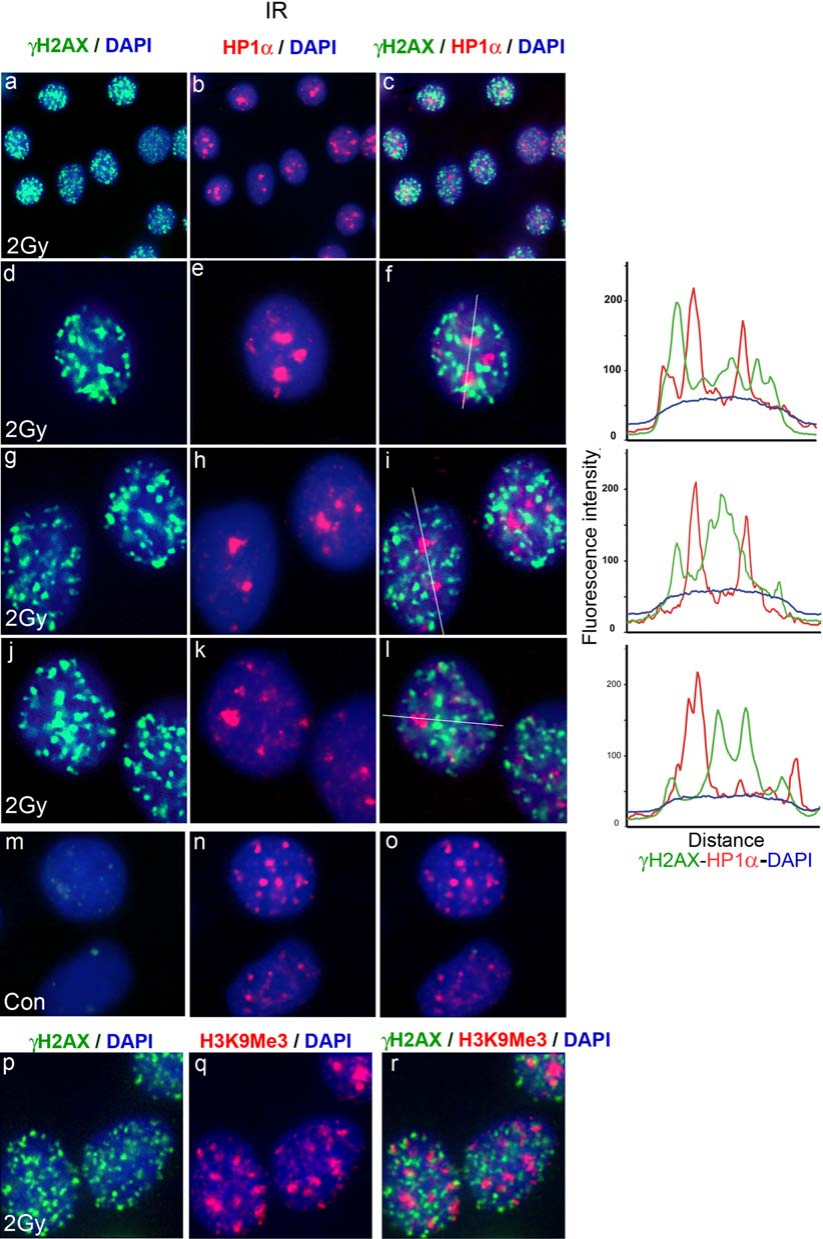
γH2AX foci induced by ionizing radiation are absent from HP1α-staining nuclear domains. MCF7 cells were fixed 30 minutes after X-irradiation (2Gy, panels a–l & p–r) and were processed for immunofluorescence for γH2AX (green) and either HP1α or H3K9Me3 (red). Panels m–o, non-irradiated cells. DNA was stained with DAPI. Panels a, d, g, j, m & p, γH2AX; panels b, e, h, k & n, HP1α; Panel q, H3K9Me3; panels c, f, I, l, o & r merged images. Overlapping red and green signals appear yellow. The top row shows a group of cells with typical appearance. Individual nuclei are shown magnified in rows 2–6. For the nuclei shown in rows 2–4, line traces were generated (shown on the right) with the line drawn through the brightest HP1α regions. For γH2AX, HP1α and H3K9Me3, images were first adjusted using levels such that fainter interfocal nuclear fluorescence was not included.

**Figure 2:**
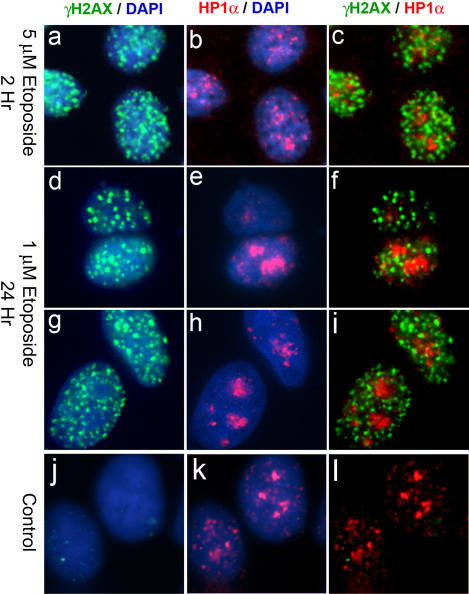
γH2AX foci generated by etoposide treatment do not appear in HP1α-staining regions in MCF7 cells. MCF7 cells were incubated in medium containing etoposide and processed as in Figure 1. Panels a–c, two hour exposure to 5 µM etoposide. Panels d–i, 24 hour exposure to 1 µM etoposide. Panels j–l, untreated cells. Cells were fixed immediately after etoposide treatment.

We previously noticed an apparently uneven distribution of γH2AX foci across the nucleus of X-irradiated MCF7 breast carcinoma cells, nuclei often containing islands free of γH2AX foci. We suspected that these γ-H2AX-free islands might include heterochromatic regions. To test this hypothesis we carried out immunofluorescence analysis for γH2AX and the heterochromatin protein HP1α in X-irradiated MCF7 cells (Fig. 1). HP1 is a highly conserved component of heterochromatin [13], [18], [19], [20], and HP1α has been reported to be concentrated in discrete nuclear regions in interphase HeLa cells, often embedding centromeres, as expected for heterochromatin [22]. Similarly, in the present study, HP1α staining was concentrated in several large nuclear domains in MCF7 cells (Fig. 1b, e, h, k&n). Prior to irradiation, γH2AX staining revealed one or two foci in most cells, as reported previously [23], [24](Fig. 1m). When cells were fixed 30 minutes after irradiation (2Gy), nuclei contained an average of 50 γH2AX foci per cell. These foci were distributed throughout the nuclei, but with apparent islands where foci were absent (Fig. 1d, g, j, p). When the γH2AX and HP1α signals were overlaid, it could be seen that the bright HP1α signals corresponded to some of the islands free of γH2AX signal (Fig. 1 third column). Line traces through selected cells emphasized this inverse correlation between γH2AX and HP1α staining. The images shown were obtained using methanol fixation, but similar results were obtained when cells were fixed with paraformaldehyde and then permeabilised. Approximately 64% of nuclei displayed no overlap between γH2AX foci and any HP1α-bright region. Cells where fewer than half of the HP1α regions contained at least one γH2AX focus made up 89% of the asynchronous cell population (see Table 1). Similar results were obtained when γH2AX foci were compared with another heterochromatin marker, Histone H3 trimethylated at lysine 9 (H3K9Me_3_, Fig 1p–r). This phenomenon was not limited to IR-generated DNA damage, as γH2AX foci appearing during treatment of MCF7 cells with the topoisomerase II poison etoposide were also largely excluded from HP1α-staining regions (Fig. 2). In this case, 60% of cells displayed no overlap between γH2AX and HP1α, while cells where less than half of the HP1α-bright regions contained at least one γH2AX focus made up 89% of the population. Similar results to those described above for MCF7 cells were also observed in mouse fibroblasts (not shown).

**Table 1:** Distribution of γH2AX foci in relation to HP1α staining in MCF7 cells treated with DNA damaging agents.

Treatment	At least one HP1α domain coincides with a γH2AX focus (mean±SD)	>50% of HP1α domains coincide with a γH2AX focus (mean±SD)
IR (2Gy)	35.7±7.4	11.1±3.6
Etoposide	39±5.9	11.6±3.9

Correlation between HP1α and H2AXγ signals was determined by eye from overlaid immunofluorescence images. In each case more than 50 nuclei were scored from 2 separate images.

### γH2AX-free islands are not simply due to nucleoli

**Figure 3:**
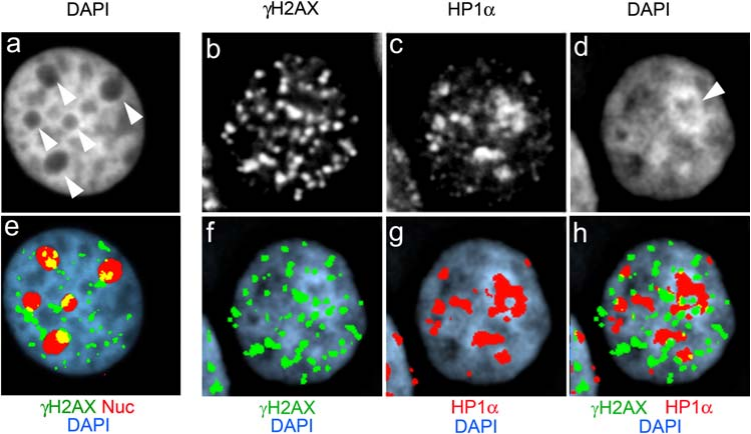
Spatial relationship between γH2AX foci, HP1α-staining heterochromatic regions and nucleoli. Panel b–d and f–h were obtained from a single nucleus fixed 30 minutes after X-irradiation (2Gy) and processed for γH2AX and HP1α immunofluorescence. Panels, a & e were obtained from a separate irradiated nucleus processed for nucleolin and γH2AX immunofluorescence. Panel a, DAPI; b, γH2AX; c, HP1α; d, DAPI; e, merged DAPI (blue) and nucleolin (red), γH2AX (green) images; f, γH2AX (green)/DAPI (blue); g, HP1α (red)/DAPI (blue); h, γH2AX/HP1α/DAPI. In panels e–h, the red and green chanels were reduced to binary images, retaining as much detail as possible, before overlaying on the DAPI image. DAPI staining was carried out under optimum conditions to reveal nuclear structure.

Nucleoli have a relatively low DNA density, and so it follows that a low frequency of DSBs would be expected per unit volume following X-irradiation. Furthermore, nucleoli are often bordered by regions of dense chromatin as judged by staining with dyes such as DAPI or TO-PRO-3. In human cells this can have the appearance of a perinucleolar rim (see Fig. 3a and Wu et al 2005 [14], for example) that partially overlaps with HP1α (see Fig. 3c, d&g). Thus, we were concerned that the apparent exclusion γH2AX foci from HP1α-staining heterochromatic regions might in fact reflect a low frequency of γH2AX foci formation within nucleoli. However, when the relative distribution of γH2AX foci and the nucleolar marker nucleolin was compared to that of γH2AX and HP1α, the γH2AX foci-free islands were primarily occupied by HP1α-staining heterochromatin and not nucleolin. Examples of these staining patterns are shown in Fig. 3a–h.

### H2AX can be phosphorylated in replicating heterochromatin

**Figure 4:**
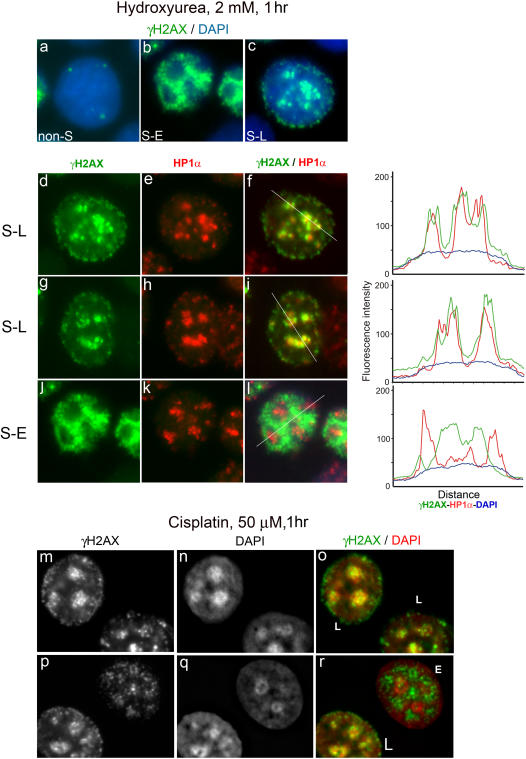
Replication stress can induce phosphorylation of histone H2AX in heterochromatin. Panels a–l, subconfluent asynchronous MCF7 cells were exposed to hydroxyurea (2 mM) for one hour immediately prior to fixation and processing for γH2AX (green) and HP1α (red) immunofluorescence. Panels a–c, representative nuclei displaying non-S phase, early to mid S-phase (S-E) and late S-phase (S-L) γH2AX staining respectively. Panels d–f & g–I, single S-L nuclei; j–l, single S-E nucleus. Panels d, g, j, γH2AX; e, h, k, HP1α; f, i, l, merged γH2AX/HP1α images. Line traces are presented on the right. Lines were drawn across the nucleus through heterochromatic (HP1α staining) regions in each case, including the DAPI channel. Panels m–r, subconfluent MCF7 cells were exposed to cisplatin (50 µM) for one hour, 38 hours after release from serum starvation. Cells were fixed immediately after cisplatin treatment and processed for γH2AX immunofluorescence. Panels m & p, γH2AX; n & q, DAPI; o & r merged γH2AX (green)/DAPI (red) images.

**Figure 5:**
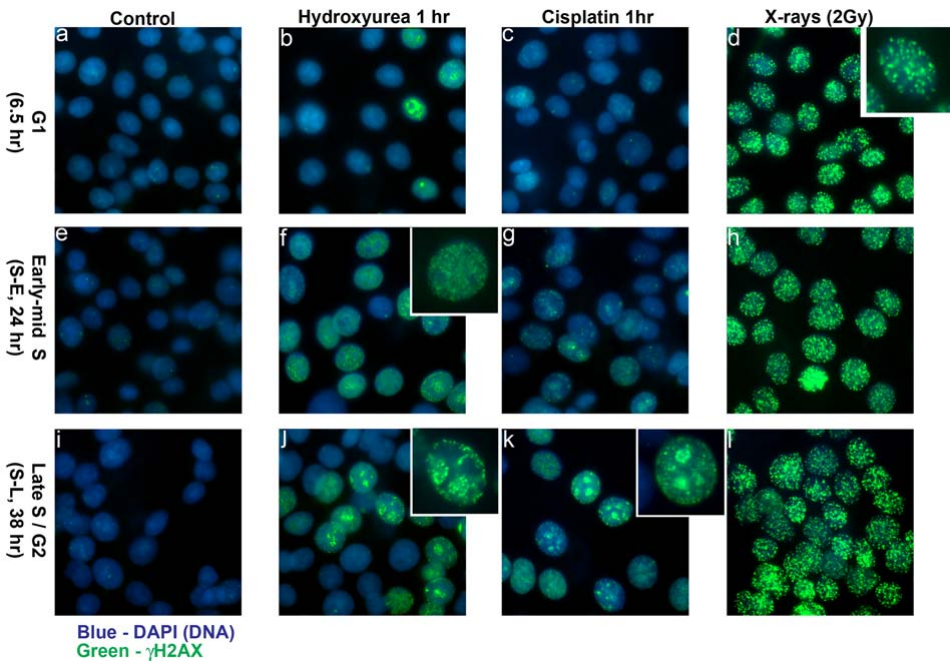
G_1_ and S-phase H2AX phosphorylation. MCF7 cells were serum starved (0.05% FCS) for 24 hours before release into medium containing 20% FCS. Cells were fixed at different times such that fixed cell populations were predominantly in G_1_, early S (S-E) or late S-phase (S-L). (6.5 hr, 24 hr and 38 hr respectively). HU or cisplatin were added to the cells, to 2 mM or 50 µM respectively, at the indicated times prior to fixation. Irradiated cells were fixed 30 minutes after irradiation.

UV irradiation or exposure to the replication inhibitor hydroxyurea (HU) results in phosphorylation of histone H2AX at sites of replication. This occurs through signaling from stalled or collapsed replication forks and is dependent on ATR [7], [25]. A feature of heterochromatin is its replication towards the end of S-phase. [14], [26], [27]. Thus, exposure of late S-phase cells to hydroxyurea would be expected to result in phosphorylation of H2AX in replicating heterochromatic regions. When asynchronous MCF7 cells were exposed to HU for 1 hour before fixation, γH2AX was either: (i) absent apart from one or two distinct foci, (ii) present throughout the nucleus in fine speckles or (iii) was clustered into large regions in the interior of the nuclei with smaller foci around the nuclear periphery (Fig. 4a–c respectively). These patterns are consistent with (i) non S-phase cells, (ii) cells in early S-phase (S-E) and lastly (iii) cells in which heterochromatic DNA is replicating in late S-phase (S-L). This interpretation was confirmed using MCF7 cells synchronized by serum starvation and release into medium containing 20% serum [28](Fig. 5,b, f, j). Notably, in S-L cells, the large γH2AX clusters coincided with the HP1α staining (Fig. 4d–i). The colocalisation of γH2AX and HP1α was examined by line traces drawn across selected nuclei, confirming the heterochromatic origin or the strongest γH2AX signals. In cells displaying the fine speckled S-phase γH2AX pattern (S-E pattern), the speckles were excluded from the HP1α staining regions (Fig 4. j–l). Similarly, treatment with the DNA crosslinking cytotoxic drug cisplatin led to phosphorylation of histone H2AX during S-phase, with γH2AX appearing in heterochromatic regions of late S-phase cells after 1 hour exposure to cisplatin (Fig. 4 m–r&Fig. 5c, g, k). Thus, H2AX is phosphorylated at sites of replication stress induced by agents such as HU and cisplatin even when those sites are within heterochromatin. Notably, when a late S-phase-enriched population of MCF7 cells were X-irradiated (Fig 5l), γH2AX foci appeared similar in overall distribution to those induced in G_1_–enriched cells (Fig 5d), but the proportion of cells exhibiting γH2AX foci overlapping HP1α domains was greater than for G1 cells (see Table 2). This suggests that during replication heterochromatic H2AX is generally more amenable to phosphorylation.

**Table 2:** Distribution of X-ray induced γH2AX foci in relation to HP1α staining during G_1_ versus late S-phase.

Phase	At least one HP1α domain coincides with a γH2AX focus (mean±SD)	>50% of HP1α domains coincide with a γH2AX focus (mean±SD)
G1	29.2±4.1	16.9±2.4
Late-S	82.2±3.2*	71.7±16.1*

MCF7 cells were serum starved (0.05% FCS) for 24 hours before release into medium containing 20% FCS. Cells were X-irradiated (2Gy) at different times and fixed 15 minutes later, such that fixed cell populations were predominantly in G_1_ or late S-phase. (6.5 hr, 38 hr respectively). Correlation between HP1α and H2AXγ signals was determined by eye from overlaid immunofluorescence images. Mean numbers of nuclei exhibiting the described coincidence of HP1α and H2AXγ signals were derived from at least two fields containing in excess of 100 nuclei.

* p<0.001 (t-test).

## Discussion

We have analyzed the distribution of γH2AX foci in relation to heterochromatin and euchromatin in the cell nucleus. γH2AX foci induced by IR were largely absent from nuclear regions containing the heterochromatin markers HP1α or H3K9Me3 in MCF7 cells. To our knowledge, this differential nuclear distribution of IR-induced γH2AX foci has not been reported previously, although re-examination of images presented in certain papers (for example [29]) shows an apparently similar pattern in mouse cells, where heterochromatin can easily be recognized as bright DAPI staining regions. Also consistent with the findings reported here, Karagiannis et al [30] reported that satellite 2 and alpha satellite-containing chromatin is resistant to the induction of γH2AX by ionizing radiation according to ChIP analysis [30]. Notably, these satellite sequences are constituents of centromeric heterochromatin. In addition, this phenomenon appears to be conserved through evolution. Kim *et al* [31] reported during the preparation of this manuscript, that in the budding yeast *Saccharomyces cerevisiae* the heterochromatic silent *HML* and *HMR* loci are resistant to γH2AX formation following the introduction a targeted DSB.

Several possible reasons can be postulated for the apparent preference of H2AX phosphorylation for the euchromatic fraction of the genome. (i) Fewer DSBs are generated in heterochromatin, (ii) histone H2AX is absent or at low abundance in heterochromatin, (iii) epigenetic or other features of heterochromatin prevent the phosphorylation of H2AX over a large enough chromatin domain to generate a detectable focus, or these features restricts access of ATM and DNA-PK, (iv) DSBs rapidly migrate to the periphery of heterochromatic regions or cause local decondensation and loss of heterochromatin features.

Starting with the first possibility, there is no consistent evidence that IR induces fewer DSBs in heterochromatin than in euchromatin. Since no intermediates other than free radicals generated following energy deposition and their interaction with the DNA molecule are involved [32], [33], [34], it appears theoretically unlikely that heterochromatin would be very refractory to DSB generation by IR. However, differences in free radical scavenging capacity between chromatin compartments could result in different sensitivities to IR. Notably, Warters and Lyons [35] showed that decondensation of chromatin in isolated nuclei by hypotonic treatment resulted in a 4.5-fold increase in the sensitivity of DNA to DSB induction as estimated by gel electrophoresis. This was presumably due to reduced protection of DNA from radical damage in decondensed chromatin associated with a reduced local concentration of histones and other proteins and molecules that scavenge free radicals. A considerable body of published work exists that compares the frequencies of radiation induced CAs originating in heterochromatin versus euchromatin, (see for example [36] and references within), but there is no consensus as to whether radiation induced CAs occur with higher or lower than expected frequencies in heterochromatin. Notably though, a recent study has shown no difference in the frequency of γ-radiation-induced chromosome breaks between the largest block of heterochromatin in the human genome (1cen-1q12) and a similarly sized euchromatic region [21]. On balance, it seems unlikely that the lack of γH2AX foci in heterochromatin could be fully accounted for by a lower sensitivity to DSB induction in these regions.

If the abundance of the H2AX histone variant was markedly lower in heterochromatin, heterochromatic DSBs would not lead to a sufficient local concentration of phospho-H2AX molecules to generate γH2AX foci that are detectable by immunofluorescence. However, this does not appear to be the case, as exposure of cells to HU during replication leads to the appearance of abundant γH2AX in heterochromatin (see Figs 4&5). Other histone modifications such as histone H3 lysine 9 trimethylation, the presence of heterochromatin-specific proteins such as HP1α, or structural features of heterochromatin may prevent access of ATM and/or DNA-PK to H2AX molecules, or may limit the extent of the domain over which H2AX is phosphorylated. However, ATR, which is responsible for H2AX phosphorylation following replication inhibition [7], appears to have access to heterochromatin at least during S-phase. Thus, ongoing replication may leave heterochromatin more amenable to DSB-induced H2AX phosphorylation. In support of this notion, a greater number of nuclei exhibit at least some overlapping γH2AX and HP1α signals when cells were irradiated in late S phase compared to G_1_ (Table 2), suggesting that transient decondensation of heterochromatin or depletion of heterochromatin proteins during replication allows H2AX phosphorylation. Further support for the role of the condensed nature of heterochromatin or its specific epigenetic and protein binding complement in preventing H2AX phosphorylation following IR comes from the use of histone deacetylase inhibitors. Prolonged exposure to low concentrations of the histone deacetylase inhibitor TSA results in reorganization of heterochromatin, characterized by increased acetylation, loss of HP1 proteins from heterochromatin and the movement of pericentromeric heterochromatin regions to the nuclear periphery [37]. Notably, Karagiannis et al [30] reported an IR-induced increase α-satellite-derived γH2AX only when cells were first exposed to TSA (0.2 µM, 72 hr). The alternative hypothesis (iv above) that the occurrence of a DSB in a heterochromatic region does result in efficient H2AX phosphorylation, but that this is coupled to local decondensation and loss of heterochromatic features seems less likely, particularly considering the data reported by Karagiannis et al. However, this possibility cannot be completely discounted in the light of data showing local chromatin decondensation at the sites of DSBs [38].

Thus, we conclude that DSBs-inducing agents fail to efficiently generate γH2AX foci in heterochromatin. The evidence discussed above suggests that this is due to the epigenetic or packaging properties of heterochromatin, preventing efficient H2AX phosphorylation. Since γH2AX is regarded as forming a platform for the recruitment or retention of other DNA repair and signaling molecules at DSBs, this implies that the processing of DSBs in heterochromatin differs from that in euchromatic regions. The differential response of heterochromatic and euchromatic compartments of the genome to DSBs will have implications for understanding the processes of DNA repair in relation to nuclear and chromatin organization.

## Materials and Methods

### Cell Culture

MCF7 cells were cultured as monolayers in RPMI 1640 medium supplemented with 10% (v/v) FCS, 100 units/mL penicillin and 100 µg/mL streptomycin. For immunofluorescence analysis, cells were grown on glass coverslips inside 6-well plates.

### Cell irradiation and drug treatment

Cells were typically cultured on glass coverslips to 50–70% confluence and X-irradiated at 2.9 Gy/min at 230 KV, 10 mA. Cells were immediately returned to the incubator for the described length of time before washing with PBS and processing for immunofluorescence. Drug treatments were carried out as described in the figure legends.

### Immunofluorescence microscopy

Coverslips were washed in PBS and cells were fixed in methanol at −20°C for 5 minutes before washing three times for 10 minutes each in PBS. Blocking was carried out overnight in KCM+T buffer [120 mM KCl, 20 mM NaCl, 10 mM Tris-HCl, pH 7.5, 0.5 mM EDTA, 0.1% (v/v) Triton X-100] containing 10% (w/v) dried milk powder and 2% (w/v) BSA. Primary and secondary antibody incubation was carried out in blocking buffer and washes were performed using KCM+T. Primary antibodies used were: mouse monoclonal anti-γH2AX (Upstate), affinity purified rabbit anti HP1α [39] and affinity purified rabbit anti-H3K9Me3 (anti Me9H3) [13]. Secondary antibodies used were Alexa Fluor® 594 goat anti-rabbit IgG and Alexa Fluor® 488 goat anti-mouse IgG (Molecular Probes). Cells were counterstained with DAPI before mounting. For Figs 1, 2 and 4a–i, DAPI was used at 1.5 µg/ml and was not washed out, resulting in uniform nuclear staining. Images were obtained using Olympus BH2-RFCA fluorescence microscope fitted with a xenon lamp and a 40× objective (DplanApo 40UV). Separate 16-bit grayscale images were recorded for DAPI, Alexa 488 and Alexa 594 using a Hamamatsu ORCA_II_ BT-1024 cooled CCD camera. Image Pro Plus software (Media Cybernetics) was used for image capture and generation of line traces. Subsequent image handling was carried out in Adobe Photoshop CS2.
